# Apogeotropic Horizontal Canal Benign Paroxysmal Positional Vertigo: Zuma e Maia Maneuver versus Appiani Variant of Gufoni

**DOI:** 10.3390/audiolres12030035

**Published:** 2022-06-19

**Authors:** Marta Alvarez de Linera-Alperi, Octavio Garaycochea, Diego Calavia, David Terrasa, Nicolas Pérez-Fernández, Raquel Manrique-Huarte

**Affiliations:** Otorhinolaryngology Department, University of Navarra Clinic, 31008 Navarre, Spain; malvarezdel@unav.es (M.A.d.L.-A.); ogaraycoche@unav.es (O.G.); dcalaviag@unav.es (D.C.); dterrasa@unav.es (D.T.); nperezfer@unav.es (N.P.-F.)

**Keywords:** vertigo, apogeotropic nystagmus, horizontal semicircular canal, repositioning maneuvers, inner ear

## Abstract

Benign paroxysmal positional vertigo (BPPV) is one of the most common disorders that causes dizziness. The incidence of horizontal semicircular canal (HSC) BPPV ranges from 5% to 40.5% of the total number of BPPV cases diagnosed. Several studies have focused on establishing methods to treat BPPV caused by the apogeotropic variant of the HSC, namely, the Appiani maneuver (App). In 2016, a new maneuver was proposed: the Zuma e Maia maneuver (ZeM), based on inertia and gravity. The aim of this study is to analyze the efficacy of App versus ZeM in the resolution of episodes of BPPV produced by an affectation of the horizontal semicircular canal with apogeotropic nystagmus (Apo-HSC). A retrospective, quasi-experimental study was conducted. Patients attended in office (November 2014–February 2019) at a third-level hospital and underwent a vestibular otoneurology assessment. Those who were diagnosed with Apo-HSC, treated with App or ZeM, were included. To consider the efficacy of the maneuvers, the presence of symptoms and/or nystagmus at the first follow up was studied. Patients classified as “A” were those with no symptoms, no nystagmus; “A/N+”: no symptoms, nystagmus present during supine roll test; “S”: symptoms present. Previous history of BPPV and/or otic pathology and calcium levels were also compiled. From the 54 patients included, 74% were women. The average age was 69. Mean follow-up: 52.51 days. In those patients without previous history of BPPV (*n* = 35), the probability of being group “A” was 63% and 56% (*p* = 0.687) when treated with App and ZeM, respectively, while being “A/N+” was 79% and 87% for App and ZeM (*p* = 0.508). Of the 19 patients who had previous history of BPPV, 13% and 64% were group “A” when treated with App and ZeM (*p* = 0.043), and 25% and 82% were “A/N+” after App and ZeM, respectively (*p* = 0.021). In conclusion, for HSC cupulolithiasis, ZeM is more effective than App in those cases in which there is a history of previous episodes of BPPV (“A”: 64% (*p* = 0.043); “A/N+”: 82% (*p* = 0.021)).

## 1. Introduction

Benign paroxysmal positional vertigo (BPPV) is one of the most common disorders that causes dizziness. It is characterized by attacks of vertigo of very short duration (seconds) caused by changes in the head position [1]. The incidence of horizontal semicircular canal BPPV (BPPV-HSC) is variable according to various authors, ranging from 5% to 40.5% of the total number of BPPV cases diagnosed [2,3,4].

The supine head roll test (SRT), or McClure–Pagnini maneuver, is the most common bedside technique used for the diagnosis of the affected side in HC-BPPV and to explain its physiopathology. In this test, the patient’s head is rotated approximately 90° to each side while lying in supine position with the head elevated at approximately 30°. During the test, the location of the otoconial debris within the canal plays a fundamental role in BPPV-HSC, inducing an ampullofugal or ampullopetal endolymph flow [1,5]. When free-floating otoconial debris is in the posterior arm of the HC and the head is turned to the affected side in SRT, according to Ewald’s second law, an ampullopetal endolymph flow will provoke a horizontal nystagmus beating towards the lowermost ear. If the head is turned to the unaffected side, an ampullofugal endolymph flow will provoke a horizontal nystagmus of lesser intensity beating towards the lowermost ear.

In the apogeotropic variant of the horizontal semicircular canal, first described by Baloh in 1995 [6], free-floating otoconial debris is floating in the anterior arm of the HC (anterior arm canalolithiasis) or attached to the cupula of the HC (cupulolithiasis) [7]. Contrary to the geotropic variant, a horizontal nystagmus beating towards the uppermost ear will be elicited if the head is turned to the affected side as a result of an ampullofugal endolymph flow, and a horizontal nystagmus of greater intensity beating towards the uppermost ear will be elicited when the head is turned to the unaffected side as a result of an ampullopetal endolymph flow.

This apogeotropic direction-changing positional nystagmus (Apo-DCPN) shows different characteristics. When transient (duration < 1 min) and without latency, it will tend towards anterior arm canalolithiasis, whereas, if the opposite is evidenced (persistent > 1 min duration, with latency), the tendency will rather be towards cupulolithiasis [8]. A small torsional component, in which the upper pole of the eye also beats towards the uppermost ear, can also be present [7]. Furthermore, cupulolithiasis can be caused by otoconial particles attached to the cupula facing the canal (C-Cup) or facing the utricle (U-Cup) [9,10]. All these factors are especially important, not only when it comes to deciding the most appropriate maneuver, but also when assessing the changes in nystagmus that may occur during follow up [11]. Other clinical tools to assess the BPPV-HSC are the “Bow and Lean” test [2] and the “Seated Supine Positioning” test [12].

For the treatment of Apo-DCPN BPPV, several maneuvers, including the modified Semont or Asprella, therapeutic headshaking, forced prolonged position, Gufoni, modified Gufoni by Appiani and, lately, the Zuma e Maia maneuver, have been introduced [4,13,14]. In 1998, Gufoni described a maneuver for BPPV-HSC as an alternative to the Lempert maneuver in elderly or overweight patients and in those with reduce mobility. Inspired by the Semont maneuver, some parts of this maneuver involve moving the patient from a seated position to a position on the right or left side, depending on the type of BPPV-HSC (geotropic/apogeotropic). In the apogeotropic form, the patient is briskly brought to the affected side, then the head is quickly inclined downwards by 45 degrees, held for 2 to 3 min and, finally, the patient is returned to the starting position [15]. In 2005, Appiani modified the Gufoni maneuver for the apogeotropic form by turning the head upwards instead of downwards (App). This change in head rotation favors the displacement of the debris by inertia and gravity because the anterior arm of the HSC is placed above the horizontal plane [16]. In 2016, Francisco Zuma e Maia proposed a new maneuver for the apogeotropic form (ZeM) based on inertia and gravity that considers the presence of free-floating otoconial debris floating in the anterior arm of the HC or debris possibly attached to the cupula facing the canal or facing the utricle. In his work, the author described a complete remission of the symptomatology and positional nystagmus in eight patients [4].

To date, there have been no other studies that analyze the effectiveness of the ZeM compared to the App. The aim of this study is to verify the benefit of the ZeM in comparison with the App in resolving the BPPV episodes produced by the apogeotropic variant of the HSC and, in particular, if previous history of BPPV influences the outcomes of each maneuver.

## 2. Methods

### 2.1. Inclusion Criteria

A retrospective, quasi-experimental study was conducted. This study took place between November 2014 and February 2019 and was performed in a third-level hospital at two different venues. During that period of time, 995 patients were treated for BPPV (all of whom were diagnosed according to current guidelines [17]), and a total of 1695 maneuvers were performed; it is important to note that the ZeM maneuver was incorporated into our strategy of treatment in 2016. Since then, the decision to assign one or other maneuver was a physician-dependent, random decision not based upon previous BPPV history or other medical factors. Inclusion criteria for this study were as follow: (1) patients of any age diagnosed with the apogeotropic variant of HC-BPPV, (2) performance of the treatment maneuver without complications on the same day of diagnosis, (3) follow up and documentation of results 2–4 weeks after the maneuver. Institutional review board approval and informed consent from all patients were obtained (RGPD 2016/679).

### 2.2. Treatment: Maneuvers

**Figure 1:**
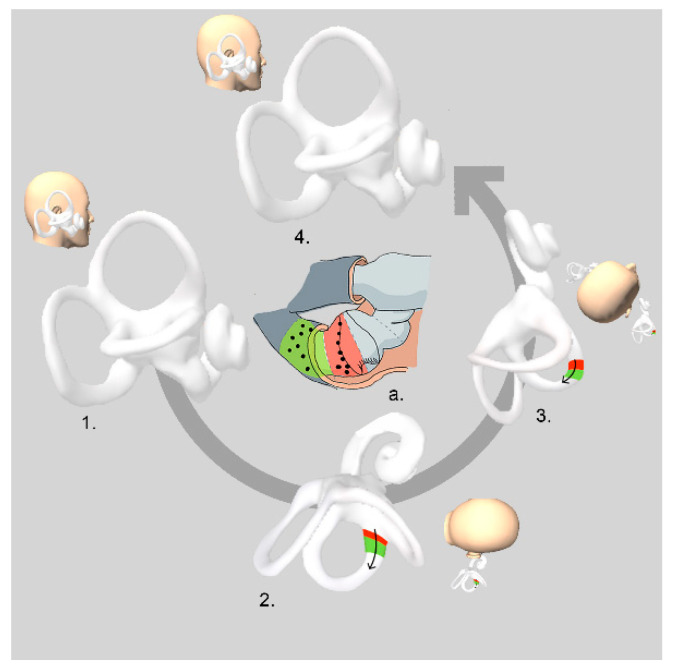
Appiani maneuver: right apogeotropic variant of HSC-BPPV. Image a: detail of horizontal canal cupulla. *Red area: otoconia attached to the cupula facing the canal (C-CUP). Green area: free-floating otoconia in the anterior arm (anterior arm canalolithiasis)*.

The Appiani maneuver [14] aims to shift the debris from the anterior into the posterior arm of the canal. Described in 2005, this maneuver is a modification of the Gufoni maneuver for the apogeotropic variant of HC-BPPV. The patient is seated in the upright position (first step), and the examiner grasps the head from behind to briskly move the patient to lie on the affected side (second step). It is expected that, with gravity (now detached by inertia), the otoconial debris facing the canal (C-Cup) and/or the free-floating otoconial debris located in the anterior arm of the HC will move towards the anterior arm and then to the bottom of the canal. Additionally, the brisk movement can detach otoconia facing the utricle (U-Cup). The position is maintained for 2 min. In the third step, the head is quickly turned upwards by 45°. This rotation induces the displacement of the otoconial debris by inertia into the posterior arm of the HSC. In the original Gufoni maneuver for the apogeotropic variant of HC-BPPV, in this third step, the head is turned downwards by 45°. According to Appiani, this modification favors the displacement of the free-floating endolymphatic debris by inertia and gravity from the anterior into the posterior arm of the HSC. Additionally, it can move the otoconial debris that might have been attached to the utricular side of the cupula towards the vestibule [18]. Finally, the patient returns to the sitting position (fourth step). In case of conversion to the geotropic variant, the treatment ends with a specific maneuver (Appiani) for that type of vertigo/nystagmus (Figure 1).

**Figure 2:**
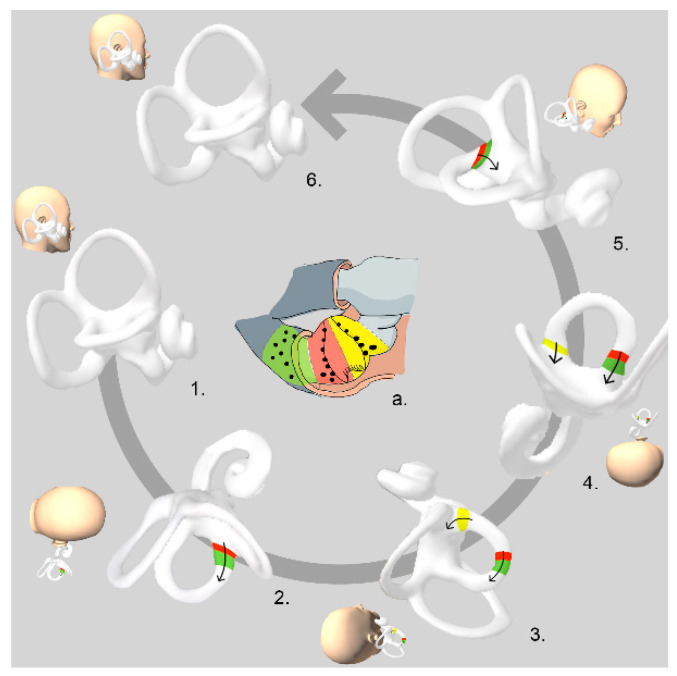
Zuma e Maia maneuver: right apogeotropic variant of HSC-BPPV: Figure a: detail of horizontal canal ampulla. *Red area: otoconia attached to the cupula facing the canal (C-Cup). Green area: free-floating otoconia in the anterior arm (anterior arm canalolithiasis*). *Yellow area: otoconia attached to the cupula facing the utricule (U-Cup)*.

The ZeM maneuver has the same initial three steps as the App; however, in this maneuver, the position is maintained for 3 min. In the third step it is also expected that otoconia attached to the utricular side of the cupula will move towards it by inertia, and/or otoconia in anterior arm will be moved towards the posterior arm by gravity [4]. The patient then moves their body into dorsal decubitus, and the head is turned 90° towards the unaffected side (fourth step) and stays in that position for 3 min. By way of this rotation, otoconial debris that could be attached to the utricular side of the cupula is removed and/or otoconial debris in the posterior arm is moved out of the HSC into the utricle. Finally, the patient’s head is tilted slightly forward in order to facilitate the particles to move towards the utricle, where they are likely to become stuck (fifth step). After 3 min, the patient returns slowly to the sitting position (sixth step) (Figure 2).

In the ZeM, the liberation of the otoliths debris is based on “inertia” and “gravity”; sudden deceleration (when the head touches the litter) is followed, in a second and third step, by rapid movements to achieve the adequate displacement of the otoliths. The fourth step strengthens the outcome of a U-Cup. Step five strengthens the result of a C-Cup or canalolithiasis of the anterior arm. The main difference between one maneuver and the other is that the ZeM can be considered as a more complete treatment since it allows the migration of the otoliths to the utricle in the case of U-Cup and through the canal (in the second and third step) in the case of C-Cup or canalolithiasis of the anterior arm [4].

### 2.3. Follow-Up

**Figure 3:**
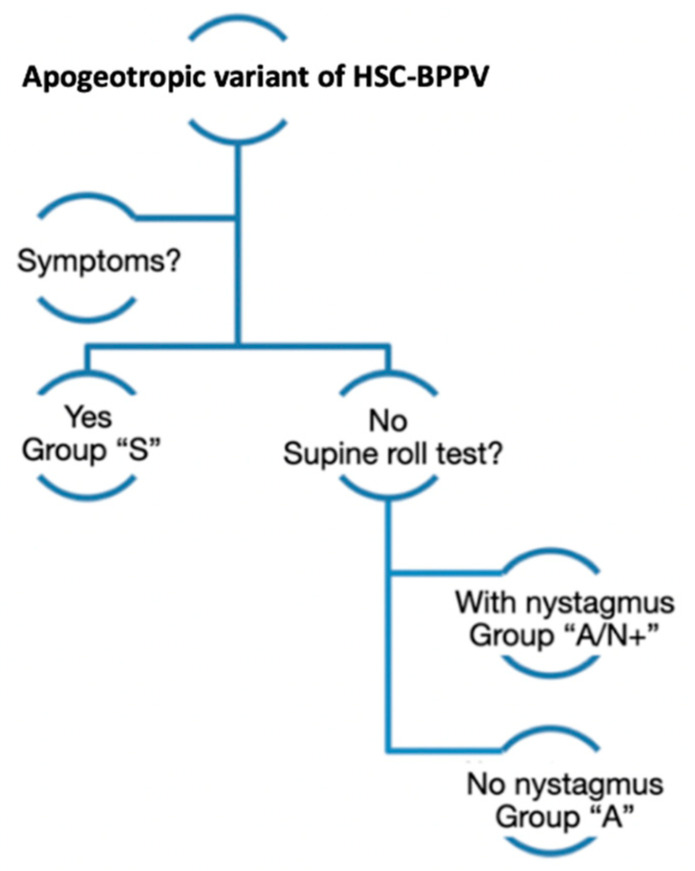
Variables studied when considering the efficacy of the maneuver. In follow-up consultation, patients diagnosed with the apogeotropic variant of BPPV-HSC were first classified in two groups according to the presence/absence of symptoms. Secondly, if patients presented symptoms, they were classified in “S” group (same clinical symptoms no examination). If this were not the case, the presence/absence of nystagmus was assessed following the supine roll test. Patients with nystagmus were classified as group “A/N+” (no symptoms but still nystagmus), while patients without it were classified as group “A” (no symptoms and no nystagmus).

When considering the efficacy of the maneuver, the variables studied were the presence of symptomatology and the presence or absence of nystagmus in supine roll test at first follow up. Based on results at follow up, patients were classified as group “A” or complete resolution: no symptoms and no nystagmus; group “A/N+”: no symptoms but still nystagmus (same nystagmus, no migration of particles) during supine roll test; group “S”: same clinical symptoms on examination (Figure 3). Patients were distributed into these groups following the previously mentioned criteria.

Moreover, the following data were also compiled: previous history of BPPV episodes prior to current episode (of any variant posterior, superior or horizontal canal cupulo- or canalithisais), previous inner ear pathology (vestibular neuritis, otosclerosis, bilateral vestibulopathy), clinical history of calcium disorders (osteoporosis, vitamin D deficiency, osteopenia) and cardiovascular risk factors.

### 2.4. Statistics

Demographic quantitative data are reported as mean (SD) or median (p25; p75) depending on distributional assumptions and n (%) in the case of qualitative data. Male and female distribution was assessed using Pearson’s chi [2]. Logistic regression was carried out to compare groups “A” and “A/N+”. Different demographic characteristics were also added to this model in order to know their possible influence on the statistical results obtained. The influence of these characteristics was reported as odds ratios (OR) with corresponding confidence interval. Distributional assumptions were checked using the Shapiro–Wilk test to assess normal distribution and quantile plots. Statistical analysis was performed using Stata 12.

## 3. Results

Out of the 54 subjects included, 40 (74%) were female. The age range was between 25 and 90 years of age, with a mean of 69. Twenty-seven patients were treated with App and 27 with ZeM, and median follow-up time was 19.5 days. Neither age (*p* = 0.511), gender distribution (*p* = 0.078), calcium disorders (*p* = 0.721) nor previous otologic disorders (*p* = 0.033) were significantly different between patients treated with one or the other maneuver. Based on the logistic regression analysis, among the facts that may influence the success rate, previous history of BPPV showed an OR of 0.083 (0.01; 0.83) (*p* = 0.034).

Within the variable calcium disorders, there were two patients who were diagnosed with osteoporosis, three with vitamin D deficiency, two with osteopenia and one with osteoporosis associated with vitamin D deficiency. Among those eight patients, six (75%) had a previous history of BPPV. Out of the patients without a diagnosis of alterations of the calcemia (*n* = 46), only 28.26% had suffered previous episodes of BPPV. Nevertheless, these results did not reach statistical significance (*p* = 0.083).

From the 27 patients treated with ZeM, 40.7% reached group “A” after one maneuver. This rate corresponded to 51.9% when analyzing the 27 patients treated with App after one maneuver. Table 1 summarizes the number of maneuvers for each group during follow up in cases that were initially either symptomatic or asymptomatic with nystagmus.

The results for each maneuver in each group of patients (with or without previous BPPV) and according to follow-up criteria appear in Table 2. In those patients without a previous history of BPPV (35 patients), the probability of reaching complete resolution during the follow up (group “A”) amounted to 63% when treated with App, while, when treated with ZeM, this percentage corresponded to 56% (*p* = 0.687). In this same group of patients, 79% represented the probability of reaching “A/N+” when treated with App, while this percentage increased to 87% in the case of patients treated with ZeM (*p* = 0.508).

**Figure 4:**
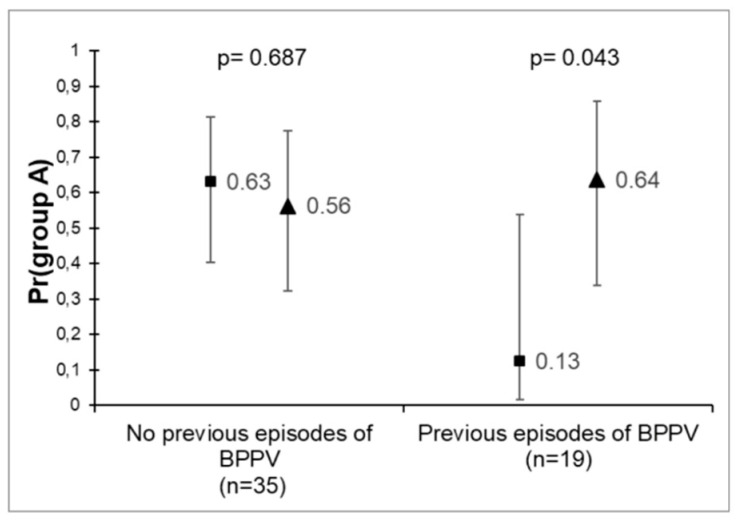
Probability of reaching “**A**” in patients with and without a history of previous episodes of BPPV after the repositioning maneuver.

On the other hand, of the 19 patients who had a previous history of BPPV, the probability of reaching complete resolution during the follow up (group “A”) amounted to 13% and 64% when treated with App and ZeM, respectively, reaching statistical significance (*p* = 0.043). Moreover, in this same group of patients, the results obtained showed an equally high probability of reaching “A/N+” for those patients treated with ZeM in comparison with those who underwent App (82% and 25%, respectively) (*p* = 0.021) (Figure 4).

## 4. Discussion

The most common peripheral cause of an apogeotropic direction-changing positional nystagmus (Apo-DCPN) is considered to be cupulolithiasis or anterior arm canalolithiasis of the HC [19]. Although different maneuvers have been proposed to address this scenario, there is no current consensus on its treatment. This is the first study comparing the therapeutic efficacies of the Appiani and the Zuma e Maia maneuvers.

Our results demonstrated a higher probability of reaching not only “A” (64%) but also “A/N+” (82%) when the patients who had previous history of BPPV were treated with ZeM, reaching statistical significance in both cases (*p* = 0.043 and *p* = 0.021, respectively). According to our results, the presence of a history of previous episodes of BPPV represents an important condition in deciding the most adequate maneuver for each patient explored.

Our study supports the theory that horizontal semicircular canal cupulolithiasis may be treated by repositioning maneuvers such as Appiani and Zuma e Maia, with a success rate of 63% and 56%, respectively (*p* = 0.687), after just a single treatment. In a review of the literature by Riga [20], the authors described a wide success rate with the Gufoni maneuver: from 22.2 to 81.3%. In this study, the author concluded that this was probably because of the difficulties in detaching otoconia from the cupula. Similar percentages of efficacy were described with the Vannucchi–Asprella maneuver [20]. Our results were similar to the study performed by Shi et al. (2018) [18], in which the authors described a remission after App in 66.2% of the patients. Interestingly, among the 14 patients in whom symptoms and/or nystagmus persisted after the maneuver (group A/N+ and group S), none of them was converted into a geotropic variant of HC-BPPV. The transition rate into a geotropic nystagmus after App also varied significantly in the literature: from 100% [16] to 25% [18]. The low incidence of conversion in our study could be explained by different reasons. Some studies suggested that U-Cup may be more frequent than C-Cup and canalithiasis in the anterior arm of the HC; therefore, if the maneuver is not effective, the persistence of the apogeotropic nystagmus after the maneuver should be more frequent than its conversion (which is only possible for C-Cup and anterior arm canalithiasis). The follow-up time was not the same for all the patients, and most of them where not examined immediately after the maneuver; consequently, we can assume that early conversion into a posterior arm canalithiasis (geotropic nystagmus) might have been missed. In this context, the persistence of the apogeotropic nystagmus in the follow-up visit might have been caused by either a U-Cup or a transformation of the posterior arm canalithiasis into a C-Cup and/or anterior arm canalithiasis, which has been described in previous studies [7]. Moreover, the underlying pathogenesis of Apo-DCPN might also have been different from BPPV-HC and difficult to distinguish, such as central positional nystagmus of probable vestibular migraine or benign recurrent vertigo; Lechner et al. (2014) [21] found striking similarities between vestibular migraine and HSC cupulolithiasis, and Shi et al. (2018) [18] concluded that migraine might be the factor that influenced the duration of spontaneous remission of Apo-DCPN.

There is scarce literature regarding the efficacy of the ZeM. When it was first described, Zuma e Maia reported a 100% success rate. Even though the study only included eight patients and the follow-up time was short [4], their results prompted our work. The lack of statistical significance found in our study could be explained by the small sample size or by the existence of other factors that might have influenced the results obtained. Among them, the potential impact of the patient’s gender should be noted. According to the results obtained here, it seems that males are four times more likely to achieve “A” without being able to reach statistical significance (*p* = 0.78). Since there is no anatomical criterion for such a difference, the explanation of this trend may be due to the greater number of female patients included in the study. This increase in the female incidence matches the results presented by other authors [22].

The pathophysiology of horizontal semicircular canal involvement in a patient showing Apo-DCPN plays a fundamental role, not only when it comes to understanding the treatment of the underlying trigger (U-Cup, C-Cup or anterior arm canalolithiasis), but also when it comes to understanding the possible benefits of one maneuver over the other. When Appiani first described his modification of the Gufoni maneuver, despite the change in the third step, the objective of the new maneuver was the same as the original: to convert an Apo-DCPN into a geotropic variant (posterior arm canalithiasis), not to resolve it. Then, how is an improvement of the symptoms and the nystagmus to be achieved with this maneuver? Shi et al. (2018) [18] suggested that the brisk movement and abrupt deceleration could probably detach the otoconia facing the utricle (U-Cup) and that the 45° upward rotation might be enough to displace the otoconia towards the vestibule. Another possibility is that the already displaced and free otoconia in the posterior arm after the maneuver are more likely to fall back into the utricle spontaneously through natural head movements [23]. Either way, according to Appiani, after performing the maneuver, the free otoconia should be in the posterior arm of the HC. Starting from this point, the last two steps of the ZeM could be considered to complement the App, favoring the adequate mobilization of the debris already located in the posterior arm towards the utricle. Moreover, the 90° upward rotation is theoretically more efficient than the 45° rotation when it comes to removing the otoconia facing the utricle (U-Cup) towards the vestibule. All of this could explain the higher “A” rate presented by the ZeM compared to the App.

**Figure 5:**
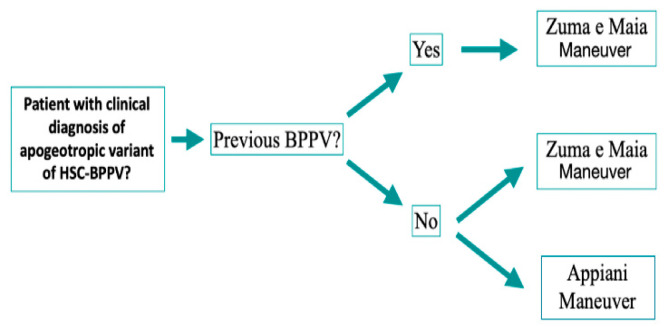
Treatment algorithm.

Unexpectedly, we found in our study that previous BPPV is itself a prognostic factor of success with the Zuma e Maia maneuver. Appiani resolved 13% of cases among those patients, whereas Zuma e Maia resolved 64% (*p* = 0.043). This could be due to the fact that recurrent BPPV in HSC is mostly associated with an underlying cupulolithiasis [24], and, while the Appiani maneuver only relocates otoconial particles attached to the cupula facing the canal, the Zuma e Maia maneuver relocates those facing the canal and those facing the utricle. Ramos et al. (2019) [7] demonstrated in 17 patients the possibility of knowing the exact location of the otoconial debris by taking into account the changes in nystagmus while performing each step of the Zuma e Maia maneuver [7]. Moreover, recurrent BPPV is also associated with a multi-channel affection [25], which includes various possible combinations of canalolithiasis and cupulolithiasis. The Zuma e Maia maneuver, by turning 180° in the horizontal plane (step 4) and by performing movements in the vertical axis (step 6), might also be able to move particles that are possibly floating in the posterior arm of the horizontal canal and/or in the vertical canals, which might not be seen during the clinical examination but could worsen the patient’s symptoms. As such, we are proposing a protocol for treatment as shown in Figure 5.

The presence of inner ear comorbidities, such as Meniere disease, and sudden hearing loss [26] can increase the risk of recurring vertigo up to 6 and 2.5 times, respectively. In our study, none of the above-described comorbidities affected our sample. Low vitamin D levels were also related to a higher rate of recurrence. Based on the composition of otoconia, it is suggested that lower levels of vitamin D and higher PTH may disrupt absorption of calcium and, thus, the resorption of calcium carbonate in the otoconia, provoking its fragmentation and dislodging in the semicircular canal [27]. Among the subjects in our study, the rates of osteoporosis and vitamin D deficiency were slightly higher in group ZeM; however, no statistical significance was seen (*p* = 0.721). The coexistence of cardiovascular diseases, such as hypertension, dyslipidemia and type I diabetes [28], was higher among patients with recurrent BPPV. BPPV patients with hypertension had a 1.51-fold higher risk of recurrence compared to those without hypertension [26]. Hypertension may cause blood vessel and circulation abnormalities leading to obstruction of inner ear circulation. Vascular stress of the anterior vestibular artery may reduce blood flow to the labyrinth and, hence, cause serious damage to the macula and otoconia detachment [29]. Hyperinsulinism may disrupt inner ear hemostasis and alter the ionic and metabolic characteristics of the stria vascularis [30]. On the other hand, hyperglycemia increases vascular resistance by inhibiting nitric-oxide-related vasodilation. Therefore, a combination of hypertension and diabetes may lead to tissue hypoxia and cochleovestibular degeneration [31]. Arterial plaque indicates early atherosclerosis. This may trigger intravascular thrombosis and cause hypoperfusion of the inner circulation [32]. In our study, cardiovascular factors did not seem to vary between treatment groups, and, unlike in previous studies, both groups had similar ages [33].

Despite all the results presented here and the non-negligible sample size, it is also important to highlight the main limitations of this study; the follow-up time was not the same for all the subjects, and the immediate effectiveness of each treatment was not assessed immediately after the maneuver. Future studies should include a time lapse between maneuvers, and a survival analysis may be performed. Moreover, although several studies have proposed various treatment options, comparisons among randomized populations are currently lacking.

## 5. Conclusions

The ZeM was shown to be more effective in the treatment of BPPV-HSC in its apogeotropic variant in patients who had previous history of BPPV. Based on our results, it is recommended that either one maneuver or the other be carried out depending on the presence or absence of a previous episode of BPPV. In the event that the patient has a history of BPPV, the most appropriate maneuver to perform is the ZeM, while, in the case of the patient not having any previous history of BPPV, the choice of one or the other of these maneuvers does not affect the effectiveness of the results obtained.

## Figures and Tables

**Table 1:** Summarizes the number of maneuvers for each group during follow up in cases that were initially either symptomatic or asymptomatic with nystagmus.

Asymptomatic Rate	Appiani	Zuma e Maia
1 maneuver	57.1%	76.9%
2 maneuvers	28.6%	19.2%
3 maneuvers	9.5%	3.8%
4 maneuvers	4.8%	

**Table 2:** Summary of outcomes after the repositioning maneuver in each group of patients with or without previous episodes of BPPV.

No Pre-BPPV	Appiani	Zuma e Maia	*p*-Value	With Pre-BPPV	Appiani	Zuma e Maia	*p*-Value
A	12	9	*0.687*	A	1	7	*0.043*
A/N+	3	5	*0.508*	A/N+	1	2	*0.021*
S	4	2		S	6	2	

A: “no symptoms, no nystagmus” A/N+: “no symptoms, nystagmus present during supine roll test; “S”: symptoms present.

## Data Availability

Not applicable.

## References

[1] Argaet E., Bradshaw A., Welgampola M. (2019). Benign positional vertigo, its diagnosis, treatment and mimics. Clin. Neurophysiol. Pract..

[2] Choung Y.H., Shin Y.R., Kahng H., Park K., Choi S.J. (2006). ‘Bow and lean test’ to determine the affected ear of horizontal canal benign paroxysmal positional vertigo. Laryngoscope.

[3] Bin Lee J., Han D.H., Choi S.J., Park K., Park H.Y., Sohn I.K., Choung Y.-H. (2010). Efficacy of the “bow and lean test” for the management of horizontal canal benign paroxysmal positional vertigo. Laryngoscope.

[4] Zuma e Maia F. (2016). New Treatment Strategy for Apogeotropic Horizontal Canal Benign Paroxysmal Positional Vertigo. Audiol. Res..

[5] Rabbitt R.D. (2019). Semicircular canal biomechanics in health and disease. J. Neurophysiol..

[6] Baloh R.W., Yue Q., Jacobson K.M., Honrubia V. (1995). Persistent direction-changing positional nystagmus: Another variant of benign positional nystagmus?. Neurology.

[7] Ramos B.F., Cal R., Brock C.M., Albernaz P.L.M., Maia F.Z.E. (2019). Apogeotropic variant of horizontal semicircular canal benign paroxysmal positional vertigo: Where are the particles?. Audiol. Res..

[8] Eggers S.D., Bisdorff A., von Brevern M., Zee D.S., Kim J.-S., Perez-Fernandez N., Welgampola M.S., Della Santina C.C., Newman-Toker D.E. (2019). Classification of Vestibular Signs and Examination Techniques: Nystagmus and Nystagmus-like Movements. J. Vestib. Res..

[9] Boleas-Aguirre M.S., Pérez N., Batuecas-Caletrío A. (2009). Bedside therapeutic experiences with horizontal canal benign paroxysmal positional vertigo (cupulolithiasis). Acta Otolaryngol..

[10] Schubert M.C. Stop the World–I Want to Get off. Vestibular SIG Newsletter. https://webcache.googleusercontent.com/search?q=cache:paFXf8Ykv_QJ:https://www.neuropt.org/docs/vsig-newsletters/bppv-special-edition-nov-2012.pdf%3Fsfvrsn%3D502ee99b_2+&cd=1&hl=zh-CN&ct=clnk.

[11] Lee S.H., Kim J.S. (2010). Benign paroxysmal positional vertigo. J. Clin. Neurol..

[12] Asprella-Libonati G. (2008). Pseudo-Spontaneous Nystagmus: A new sign to diagnose the affected side in Lateral Semicircular Canal Benign Paroxysmal Positional Vertigo. Acta Otorhinolaryngol. Ital..

[13] Yamanaka T., Sawai Y., Murai T., Okamoto H., Fujita N., Hosoi H. (2014). New treatment strategy for cupulolithiasis associated with benign paroxysmal positional vertigo of the lateral canal: The head-tilt hopping exercise. Eur. Arch. Otorhinolaryngol..

[14] Han K., Lee J., Shin J.E., Kim C.H. (2021). Treatment Efficacy of Forced Prolonged Position After Cupulolith Repositioning Maneuver in Apogeotropic HSCC BPPV. Ear Nose Throat J..

[15] Gufoni M., Mastrosimone L., di Nasso F. (1998). Repositioning maneuver in benign paroxysmal positional vertigo of the horizontal semi-circular canal. Acta Otorhinolaryngol. Ital..

[16] Appiani G.C., Catania G., Gagliardi M., Cuiuli G. (2005). Repositioning Maneuver for the Treatment of the Apogeotropic Variant of Horizontal Canal Benign Paroxysmal Positional Vertigo. Otol. Neurotol..

[17] Von Brevern M., Bertholon P., Brandt T., Fife T., Imai T., Nuti D., Newman-Toker D. (2015). Benign paroxysmal positional vertigo: Diagnostic criteria. J. Vestib. Res..

[18] Shi T., Yu L., Yang Y., Wang Y., Shao Y., Wang M., Geng Y., Shi Z., Yin X. (2018). The effective clinical outcomes of the Gufoni maneuver used to treat 91 vertigo patients with apogeotropic direction-changing positional nystagmus (apo-DCPN). Medicine.

[19] Bhattacharyya N., Gubbels S.P., Schwartz S.R., Edlow J.A., El-Kashlan H., Fife T., Holmberg J.M., Mahoney K., Hollingsworth D.B., Roberts R. (2017). Clinical Practice Guideline: Benign Paroxysmal Positional Vertigo (Update). Otolaryngol. Head Neck Surg..

[20] Riga M., Korres S., Korres G., Danielides V. (2013). Apogeotropic variant of lateral semicircular canal benign paroxysmal *positional* vértigo: Is there a correlation between clinical findings, underlying pathophysiologic mechanisms and the effectiveness of repositioning maneuvers?. Otol. Neurotol..

[21] Lechner C., Taylor R.L., Todd C., MacDougall H., Yavor R., Halmagyi G.M., Welgampola M.S. (2014). Causes and characteristics of horizontal positional nystagmus. J. Neurol..

[22] Vibert D., Kompis M., Hausler R. (2013). Benign paroxismal postional vértigo in older women may be related to osteoporosis and osteopenia. Ann. Otol. Rhinol. Laryngol..

[23] Korres S., Balatsouras D.G., Kaberos A., Economou C., Kandiloros D., Ferekidis E. (2002). Occurrence of semicircular canal involvement in benign paroxysmal positional vertigo. Otol. Neurotol..

[24] Choi S.J., Lee J.B., Lim H.J., Park H.Y., Park K., In S.M., Oh J.H., Choung Y.-H. (2012). Clinical Features of Recurrent or Persistent Benign Paroxysmal Positional Vertigo. Otolaryngol. Head Neck Surg..

[25] Shim D.B., Song C.E., Jung E.J., Ko K.M., Park J.W., Song M.H. (2014). Benign paroxysmal positional vertigo with simultaneous involvement of multiple semicircular canals. Korean J. Audiol..

[26] Zhu C.T., Zhao X.Q., Ju Y., Wang Y., Chen M.M., Cui Y. (2019). Clinical Characteristics and Risk Factors for the Recurrence of Benign Paroxysmal Positional Vertigo. Front. Neurol..

[27] Al Garni M.A., Mirza A.A., Althobaiti A.A., Al-Nemari H.H., Bakhsh L.S. (2018). Association of benign paroxysmal positional vertigo with vitamin D deficiency: A systematic review and meta-analysis. Eur. Arch. Otorhinolaryngol..

[28] Yoda S., Cureoglu S., Yildirim-Baylan M., Morita N., Fukushima H., Harada T., Paparella M.M. (2011). Association between Type 1 Diabetes Mellitus and Deposits in the Semicircular Canals. Otolaryngol. Head Neck Surg..

[29] Babac S., Djeric D., Petrovic-Lazic M., Arsovic N., Mikic A. (2014). Why do treatment failure and recurrences of benign paroxysmal positional vertigo occur?. Otol. Neurotol..

[30] Webster G., Sens P.M., Salmito M.C., Cavalcante J.D.R., dos Santos P.R.B., da Silva A.L.M., de Souza C.F. (2015). Hyperinsulinemia and hyperglycemia: Risk factors for recurrence of benign paroxysmal positional vertigo. Braz. J. Otorhinolaryngol..

[31] D’Silva L.J., Staecker H., Lin J., Sykes K.J., Phadnis M.A., McMahon T.M., Connolly D., Sabus C.H., Whitney S.L., Kluding P.M. (2016). Retrospective data suggests that the higher prevalence of benign paroxysmal positional vertigo in Individuals with type 2 diabetes is mediated by hypertension. J. Vestib. Res..

[32] Wada M., Takeshima T., Nakamura Y., Nagasaka S., Kamesaki T., Kajii E. (2016). Carotid plaque is a new risk factor for peripheral vestibular disorder: A retrospective cohort study. Medicine.

[33] De Stefano A., Dispenza F., Suarez H., Perez-Fernandez N., Manrique-Huarte R., Ban J.H., BeomKim M., Strupp M., Feil K., Oliveira C.A. (2014). A multicenter observational study on the role of comorbidities in the recurrent episodes of benign paroxysmal positional vertigo. Auris Nasus Larynx.

